# T1-refBlochi: high resolution 3D post-contrast T1 myocardial mapping based on a single 3D late gadolinium enhancement volume, Bloch equations, and a reference T1

**DOI:** 10.1186/s12968-017-0375-1

**Published:** 2017-08-18

**Authors:** Chenxi Hu, Albert J. Sinusas, Steffen Huber, Stephanie Thorn, Mitchel R. Stacy, Hamid Mojibian, Dana C. Peters

**Affiliations:** 1 Department of Radiology and Biomedical Imaging, Yale School of Medicine, New Haven, CT 06520 USA; 2 Department of Internal Medicine (Cardiology), Yale School of Medicine, New Haven, CT 06520 USA

**Keywords:** 3D cardiac T1 mapping, Late gadolinium enhancement, Single-TI T1 mapping, Left atrial fibrosis

## Abstract

**Background:**

High resolution 3D T1 mapping is important for assessment of diffuse myocardial fibrosis in left atrium or other thin-walled structures. In this work, we investigated a fast single-TI 3D high resolution T1 mapping method that directly transforms a 3D late gadolinium enhancement (LGE) volume to a 3D T1 map.

**Methods:**

The proposed method, T1-refBlochi, is based on Bloch equation modeling of the LGE signal, a single-point calibration, and assumptions that proton density and T2* are relatively uniform in the heart. Several sources of error of this method were analyzed mathematically and with simulations. Imaging was performed in phantoms, eight swine and five patients, comparing T1-refBlochi to a standard spin-echo T1 mapping, 3D multi-TI T1 mapping, and 2D ShMOLLI, respectively.

**Results:**

The method has a good accuracy and adequate precision, even considering various sources of error. In phantoms, over a range of protocols, heart-rates and T1 s, the bias ±1SD was -3 ms ± 9 ms. The porcine studies showed excellent agreement between T1-refBlochi and the multi-TI method (bias ±1SD = −6 ± 22 ms). The proton density and T2* weightings yielded ratios for scar/blood of 0.94 ± 0.01 and for myocardium/blood of 1.03 ± 0.02 in the eight swine, confirming that sufficient uniformity of proton density and T2* weightings exists among heterogeneous tissues of the heart. In the patients, the mean T1 bias ±1SD in myocardium and blood between T1-refBlochi and ShMOLLI was -9 ms ± 21 ms.

**Conclusion:**

T1-refBlochi provides a fast single-TI high resolution 3D T1 map of the heart with good accuracy and adequate precision.

**Electronic supplementary material:**

The online version of this article (doi:10.1186/s12968-017-0375-1) contains supplementary material, which is available to authorized users.

## Background

T1 mapping has been recognized in recent years as an important potential biomarker for tissue characterization in cardiovascular magnetic resonance (CMR). Several clinical studies have shown that post-contrast T1 values, the focus of this paper, are significantly shorter in the left ventricular (LV) myocardium for several cardiac diseases, beyond myocardial infarction [1, 2], including dilated cardiomyopathy [3, 4], amyloidosis [5], and heart failure [6]. Moreover, post-contrast T1 enables calculation of extracellular volume (ECV) fraction, which is a robust and sensitive biomarker to diffuse myocardial fibrosis, undetectable by late gadolinium enhancement [7].

A number of breath-hold CMR T1 mapping sequences have been proposed, including MOdified Look-Locker Inversion recovery (MOLLI) (or shortened MOLLI- ShMOLLI), saturation recovery single-shot acquisition (SASHA), and SAturation Pulse Prepared Heart RAte independent Inversion-REcovery (SAPPHIRE) [8–12]. Other methods, including Accelerated and Navigator-Gated Look-Locker IMaging (ANGIE) and an interleaved T1/T2 method [11, 13], provide higher in-plane spatial resolution and use navigator-gating. However, all these techniques focus on 2D T1 mapping.

For the LV, although 2D T1 mapping may provide sufficient spatial resolution, errors may arise due to partial-voluming [14]. For example, high resolution T1 mapping of the LV has an important role in mapping the morphology of arrhythmic scar, for detection of grey zone. T1 mapping might be more accurate and reproducible than LGE-based grey zone detection for identifying intermingled viable and nonviable myocytes [15, 16]. For other thin-walled chambers, such as left atrium and right ventricle (RV), low-resolution T1 mapping has very limited value [17–19]. Even so, diffuse fibrosis of the RV [20] has been observed in subjects with pulmonary hypertension using a high resolution 2D method, and further increases in resolution would benefit assessment of the RV.

In the left atrium, histology has shown that fibrosis is often diffuse with a regional pattern [21, 22]. Some of this diffuse fibrosis can be detected using high resolution LGE, because of its extensiveness (ECV > 50%) and regionality, i.e. with the left atrial wall enhanced on LGE compared to blood, and compared to other atrial wall regions. However, atrial fibrosis, including post-ablation scar, is not as demarcated and dramatic as is scar found in LV infarction. Therefore, a more accurate measure of atrial fibrosis is critical, in order to perform the necessary studies determining the prognostic value of atrial fibrosis for later atrial fibrillation, stroke, heart-failure and mortality. All of these studies are ongoing [23–25], and more quantitative metrics of atrial fibrosis is crucial in this effort. Similar to measurement of grey zone in infarction, the choice of thresholding for including atrial fibrosis and scar is uncertain. No threshold choice is ideal. The optimal choice depends on the scan parameters, and time post injection of imaging. This leads to inaccurate and time-consuming segmentation of atrial fibrosis and scar. Measurement of T1 and ECV in the atrium could replace measurement of percent fibrosis and may better correlate to atrial volumes, ejection fraction, strain, age, and risk factors of atrial fibrillation. This paper presents the early steps in that effort.

However, 3D cardiac T1 mapping remains challenging due to the excessively long scan time. The imaging time of a single 3D CMR sequence, such as 3D high resolution LGE, is already long (around 5 min), due to the increased size of k-space, cardiac gating, and navigator-gating. A T1 mapping sequence usually triples or even quadruples the imaging time compared to a single T1-weighted sequence, due to the multiple T1 weightings (TIs) needed, making the scan time formidably long for clinical utilization. Recently, several 3D cardiac T1 mapping methods have been proposed, employing different strategies to reduce the imaging time [26–29]. Coniglio et al. [26] used two 3D inversion recovery measurements with different inversion times (TIs). Nordio et al. [27] developed a 3D SASHA technique with a relatively small number of slices and radial k-space shutters. Others [28, 29] employed k-space undersampling with a compressed sensing reconstruction to measure native and post-contrast T1, respectively.

Among various T1 mapping methods, several recent studies [30–34] use a priori knowledge of spin history to improve quantification. In a similar spirit, we propose a novel fast 3D high resolution T1 mapping method that permits acquisition of a 3D T1 map using only a single TI. The method, termed T1-refBlochi (which uses a reference signal and Bloch equations), directly transforms a 3D high resolution LGE image to a 3D T1 map. To make the transformation, Bloch equation modeling of the signal in 3D LGE is used to establish a one-to-one relationship in each voxel between the signal and the underlying T1. The combined proton density and T2* weighting in the LGE signal is unknown, but assumed to be relatively uniform in the heart, so that a single-point calibration, using a reference T1 and its corresponding signal in the LGE image, is sufficient by providing an estimate of the unknown weight in a single tissue. The image intensity is then transformed to T1 voxel-by-voxel, based on the single-point estimate of the proton density and T2* weighting. The proposed method is very fast, considering only a single TI is needed; however, several confounding factors exist and are analyzed.

In this work, we investigate the performance of this novel technique with mathematical analysis, simulations, phantom experiments, and in vivo porcine studies. We characterized the accuracy and precision of the method under several confounding factors, including an inaccurate reference T1, spatial variation of proton density and T2*, inaccuracy in time between inversions (R-R time), an imperfect inversion, with both mathematical derivations and Bloch equation simulations.

We validate the performance of the method based on phantom experiments and in vivo porcine studies. Since in vivo validation of 3D T1 mapping using a 2D T1 mapping (e.g. MOLLI) is difficult due to differences in partial-voluming, we compared the proposed technique to a 3D multi-TI method of the same resolution, which (unlike MOLLI) can provide a map of the combined proton density and T2* weightings; this is needed to determine the uniformity of these weightings. Finally, the proposed method was compared to ShMOLLI in a small cohort of 5 patients as a proof-of-concept to demonstrate the clinical feasibility of this technique.

## Methods

### Theory

**Fig. 1:**
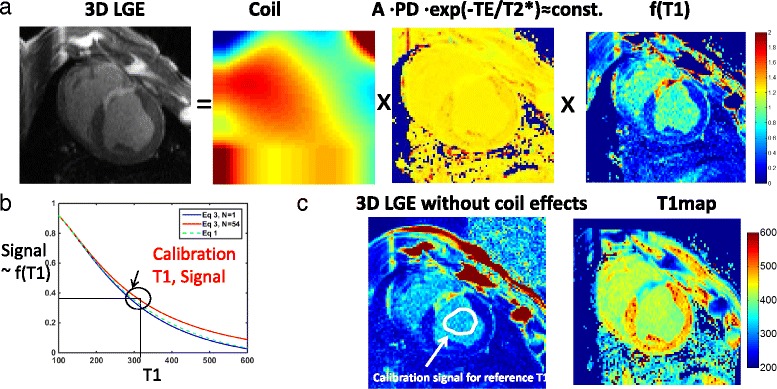
**a** The 3D LGE image is composed of weightings due to the coil, proton density (PD), and T2*, in addition to the predominant T1 weighting, f(T1). **b** The signal vs. T1 curve plots the steady-state Mz, based on the accurate model in Eq. 3 (*blue* and *red*); the approximated model of Eq. 1 (*green* dots) is also shown. N (number of views per segment) varies from 1 (*blue*) to 54 (*red*) to illustrate the difference between the accurate and the approximated models. Other parameters were: TR /*α*/TI/RR = 5.0 ms/15°/320 ms/950 ms. **c** The T1-refBlochi method removes coil-sensitivity with image processing, and assumes that T2* and PD weightings are constant. Then the underlying T1-weighting can be modeled using exact Bloch equations, and a T1 map can be extracted with a single calibration point on the T1 vs. signal curve—the reference T1

The LGE signal is composed of weightings due to coil sensitivity, proton density (PD) and T2*, and T1-weighting (Fig. 1a). We have developed an analytic expression for the signal from 3D LGE. The LGE sequence is an inversion recovery prepared FLASH sequence. During each RR interval, the sequence applies a number of small flip angle pulses (*α* pulses) for data acquisition following the inversion pulse and subsequent inversion time (TI). For a simple IR GRE sequence, which consists of repeated blocks of an IR pulse, a TI time, a 90° excitation pulse, and another regrowth period (RR-TI), there is a well-known formula for the steady state longitudinal magnetization M_zss_ [35]:

1 $$ {M}_{zss}\left({T}_1,{M}_0, TI, RR\right)={M}_0\left(1-2{e}^{-\frac{TI}{T_1}}+{e}^{-\frac{RR}{T_1}}\right) $$

where M_zss_ is the magnetization just prior to the 90° excitation pulse, TI is the inversion time, RR is the RR-interval. *M*_0_ is a complex scaling factor that includes impacts from proton density, T2* weighting, and coil sensitivity, and is represented by.

2 $$ {M}_0=A\cdotp C\cdotp PD\cdotp \exp \left(-\frac{TE}{T_2^{\ast }}\right) $$

where A is a spatially invariant constant, C is coil sensitivity, PD is proton density, and the last term is the T2* weighting. Eq. 1 has often been used to approximate the signal in the LGE sequence [36, 37], but the model ignores the perturbation of the magnetization by the *α* pulse train. To improve the model accuracy, we derived a more complex expression for M_zss_ (just prior to 1st α pulse) that includes the perturbation from the *α* pulse train:

3 $$ {M}_{zss}\left({T}_1,{M}_0, TI, RR,\alpha, TR,N\right)={M}_0\left(\frac{1-2{e}^{-\frac{TI}{T_1}}+{pe}^{-\frac{RR}{T_1}}}{1+{\left(\mathit{\cos}\left(\alpha \right)\right)}^N{e}^{-\frac{RR}{T_1}}}\right) $$

where *N* is the number of *α* pulses in the train (the views per segment), *α* is the flip angle, TR is the time between two *α* pulses, and


is referred to as the perturbation coefficient (*p* ≥ 1). The derivation of the Eq. 3 is in the Appendix A. If *α* = *π*/2, *N* = 1, then *p* = 1, and Eq. 3 reduces to Eq. 1; otherwise, M_zss_ increases due to use of the *α* pulse train. Fig. 1b shows the difference between the two models, across a spectrum of T1 s when N increases from 1 to 54.

### T1-refBlochi

Most T1 mapping methods are based on multiple measurements of the image at different TIs. However, multiple TIs may be nearly impractical for clinical usage in high resolution 3D T1-mapping because of exceedingly long acquisition time. In this section, we demonstrate that with a single measurement it is also possible to generate a T1 map, given a known reference T1 for a single tissue, and some reasonable assumptions about proton density and T2* uniformity in the heart. Fig. 1 shows the basic idea of T1-refBlochi. The prerequisite for this method is a one-to-one relationship between the LGE signal and the underlying T1, which is true as long as the signal-T1 curve, defined in Eq. 3, is monotonic in the post-contrast T1 range. This condition is guaranteed if the TI is sufficiently large so that the entire post-contrast T1 range is within the descending part of the LGE signal-TI curve (cf. Fig. 1). Once the one-to-one relationship between signal and T1 is established, the only obstacle in solving Eq. 3 for T1 is *M*_0_, which is usually unknown and requires multiple TIs to jointly estimate the two variables via voxel-by-voxel curve fitting. However, the requirement of multiple TIs can be relieved if *M*_0_ is spatially uniform in the heart region. In this case, since *M*_0_ is only a scalar, it can be estimated by using a reference signal, with a known T1, which is essentially a single-point calibration in the signal-T1 curve (cf. Fig. 1b). In practice, this can be achieved by using a T1 standard, placed around the patient’s body, or using blood (Fig. 1c), whose T1 can be obtained by a 2D breath-hold scan (TI-scout) or a MOLLI sequence, for example. The blood pool is a reasonable choice for an endogenous reference. Firstly, it has a homogeneous T1—so is easily measured using ROIs. Secondly, its T1 is a value intermediate between myocardium and scar. Dividing the reference signal by the model signal yields the scaling factor *M*_0_ of Eq. 3. Then for each signal intensity in each slice, a T1 value can be determined based on Eq. 3. A grid search over a practical T1 range was used to generate the T1 value at each voxel. In our implementation, 100 ms–800 ms was used as the T1 range and 2.5 ms was used as the grid search density.

### M_0_ heterogeneity

The spatial variation of M_0_ in the region of heart is assumed to be minor, after removing the effects of coil sensitivity. This assumption permits the single-TI T1 mapping based on a single-point calibration. Below, literature data justifying this assumption is shown and the detrending method to remove the coil sensitivity in this paper is introduced. In results, we directly measure M_0_ and demonstrate that this assumption is reasonable in a study of eight swine.

T2* differences in myocardium and blood provide only minor weightings. It’s known that the native T2* of blood is about 200 ms [38], while the T2* of myocardium is 50 ms [39], although lower in iron overload disease [40], and with hemorrhage [41]. Contrast agent will reduce these T2*s [38], differently in whole blood vs. tissue with and without infarction. Very recent data show that 5 to 10 min after injection of contrast, myocardial T2* decreases by 4 ms in normal myocardium, and 7 ms in regions of chronic infarct, compared to pre-contrast values [42]. An estimate of how T2* variation will effect M_0_ (Eq. 2) for T2*s of myocardium ranging from 50 to 10 ms, and T2*s of blood from 200 to 10 ms yields a worst case effect of 10% for a TE of 2 ms.

Secondly, the proton density, or its analogue, water content, of blood and myocardium are similar, although data is limited. Myocardium has a water content of 80% [43]. The plasma which is (1-hematocrit) of blood contains 93% water [43]. The erythrocytes make up the rest of blood, and are 64% water by weight [44]. This yields, for a HCT of 45%, a water content of about 80% for blood. A study of normal, ischemic and occluded and reperfused (i.e. highly edematous) myocardium showed that the water content was 80, 81 and 84% respectively [45]. Acutely infarcted tissue was measured by Higgins et al. to have an elevated water content of 79% vs. 76% for normal myocardium [46]. In chronic fibrotic myocardium, the T2 is similar to normal myocardium, suggesting that water content returns to normal values [47]. This literature data suggests that proton density weighting is a minor source of error (up to 4%).

Finally, the signal intensity variation caused by coil sensitivity is removed by a custom post-processing algorithm before the actual T1 mapping (see Fig. 1a). A number of post-processing methods for removal of the slowly-varying coil sensitivity has been proposed, mainly in brain or musculoskeletal imaging [48, 49] and cardiac perfusion imaging [50]. Similarly to these methods, our method uses a non-contiguous volume of interest (VOI) placed in the (presumably homogeneous) blood pool to extract a 3D coil sensitivity map. A cubic polynomial fit was performed to extract the slowly-varying coil profile from the noisy signal in the VOI. The 3D trend was then extrapolated based on the cubic polynomial parameters to cover the heart. Finally, the 3D trend was divided out of the volume to remove the coil sensitivity effect.

### Accuracy of T1-refBlochi

The accuracy of T1-refBlochi is influenced by several factors, including inaccurate reference T1 estimate, M_0_ heterogeneity, inaccurate RR duration estimate, and imperfect inversion. Other minor factors may also exist, such as off-resonance, heart rate, and flow; these we briefly address in Discussion.

The reference T1 estimated by a separate 2D scan, such as TI-scout or MOLLI, can be biased for example due to T1 drift. The inaccurate T1 estimate of the reference tissue leads to a biased M_0_ estimate, which later leads to a biased estimate of T1 in every tissue. M_0_ heterogeneity due to proton density and T2* spatial variation also causes a biased M_0_ estimate. However, this effect should be small (≤10%) based on the previous literature and our observation, as described above. In practice, we used the average RR recorded in the DICOM header when evaluating Eq. 3. However, the RR duration varies in vivo, and the RR value present during collection of central k-space data determines the image contrast. Therefore, some T1 error could result from inaccurate LGE modeling due to the inaccurate RR estimate. Imperfect inversion is generally present in any inversion-based T1 mapping methods [51]. Like the inaccurate RR estimate, imperfect inversion generates errors in the LGE signal modeling (Eq. 3) that assumes a perfect inversion.

The impact of all these four major factors can be broadly divided into two categories. The first category has bias in the estimate of M_0_, which later causes error in the T1 estimate. The second category uses an inaccurate modeling of the LGE signal, which then causes biased T1 estimate. The T1 error in both categories can be mathematically derived based on Eq. 3. Here, the formulae are given while the specific derivation is shown in Appendix B. In the simulation studies, we verified the accuracy of these formulae by comparing them to direct simulation of the bias. To begin the analysis, let.

5 $$ f\left({T}_1\right)=\frac{1-2{e}^{-\frac{TI}{T_1}}+{pe}^{-\frac{RR}{T_1}}}{1+{\left(\cos \left(\alpha \right)\right)}^N{e}^{-\frac{RR}{T_1}}} $$

represent the T1-weighting, hence Eq. 3 can be rewritten as

6 $$ {M}_{zss}\left({T}_1,{M}_0, TI, RR,\alpha, TR,N\right)={M}_0f\left({T}_1\right) $$

In Category 1, the estimate of M_0_ from the single-point calibration is wrong, presumably by a factor of *δ*, i.e., ${\widehat{M}}_0=\delta {M}_0$, where ${\widehat{M}}_0$ represents the estimated M_0_. The resultant bias of T1, Δ*T*_1_, is then approximately given by (see Appendix B).

7 $$ \Delta {T}_1\approx \frac{\left(1-\delta \right)f\left({T}_1\right)}{\delta {f}^{\prime}\left({T}_1\right)} $$

where *f* ^′^(·) is the first-order derivative of *f*(·) with respect to T1. For a fixed LGE protocol, both *f*(*T*_1_) and *f* ^′^(*T*_1_) for each T1 is known, so the bias of T1 estimate can be approximately evaluated given the bias in the estimate of *M*_0_.

In Category 2, not only is M_0_ incorrectly estimated, but the function *f*(·) is also slightly biased compared to the true function, here denoted by *f* ^*True*^(·). In this case, the resultant bias of T1, Δ*T*_1_, is given by.

8 $$ \Delta {T}_1\approx \frac{f^{True}\left({T}_1\right)-\updelta f\left({T}_1\right)}{{\updelta f}^{\prime}\left({T}_1\right)} $$

Hence, if *f* ^*True*^(·) is given, the bias of T1 can also be predicted by this equation.

### Precision of T1-refBlochi

For MOLLI or other multi-TI T1 mapping approaches, the precision depends on a number of factors, including SNR of the raw images, number of TIs, and number of parameters in the curve fitting [14]. For T1-refBlochi, there is only one parameter, i.e. T1, and only one measurement, i.e. the LGE signal, in the curve fitting at each voxel. The precision thus depends on SNR of the LGE image and the first-order derivative of the signal-versus-T1 function, i.e. the absolute value of *f* ^′^(·) at the underlying T1. SNR of blood for 3D LGE is typically low, in the range of 10–20. ∣*f* ^′^(·)∣ is a monotonically decreasing function, making precision of the T1 estimate better for smaller T1 s and worse for larger T1 s. The precision for blood SNRs of 10 and 15 was explored in the simulations.

### Simulations

Simulations were performed to verify Eq. 7 and Eq. 8 and to show the accuracy and precision of T1-refBlochi under various sources of error. To simulate T1-refBlochi, LGE signal of T1 ranging from 200 ms to 600 ms was generated based on Eq. 3. The parameters of the LGE sequence in the simulations were TR/ *α*/TI/RR/*N* = 5 ms/15°/300 ms/900 ms/33. The reference tissue has a T1 of 280 ms, and M_0_ of 1. This reference T1 was chosen to simulate the blood T1, which was used in the in vivo studies as the reference.

### Accuracy

We performed simulations to study the impact of the four major sources of error in the accuracy of T1 estimate. To simulate the impact of biased reference T1 estimate, two biased reference T1 s, 250 ms (true reference T1 – 30 ms) and 310 ms (true reference T1 + 30 ms), were used. The single-point calibration generated a biased M_0_ estimate, which was used to perform T1 mapping. To simulate spatial variation of M_0_, we used M_0_ of 1.1 or 0.9 (±10% change from the reference M_0_) for all non-reference tissue. To simulate a biased RR duration, we used a nominal RR of 930 ms and 870 ms (true RR ± 30 ms) in T1-refBlochi. Finally, to simulate the imperfect inversion, we used an inversion factor of 0.92 to simulate the true LGE signal. This inversion factor is the worst-case scenario for the hyperbolic secant RF pulse we used, according to the investigation in [51]. For the imperfect inversion case, the LGE signal was generated based on the following formula:

9 $$ {f}_{\beta}\left({T}_1\right)=\frac{1-\left(1+\beta \right){e}^{-\frac{TI}{T_1}}+{p\beta e}^{-\frac{RR}{T_1}}}{1+\beta {\left(\mathit{\cos}\left(\alpha \right)\right)}^N{e}^{-\frac{RR}{T_1}}} $$

where *β* represents the inversion factor. The derivation of the equation is similar to that of Eq. 3. *β* = 0.92 in the simulation.

For all the simulations, we calculated the bias of T1 estimate relative to the true T1 in the range of T1 from 200 ms to 600 ms, covering the typical post-contrast T1 range. To verify Eq. 7 and Eq. 8, we also calculated the bias of T1 directly from these equations.

### Precision

The precision of T1-refBlochi was studied using Monte-Carlo simulations [14]. Specifically, 10,000 Monte-Carlo simulations of LGE signal were employed and T1-refBlochi was applied to each of them for T1 ranging from 200 ms to 500 ms. The parameters of the LGE protocol was TR/ *α*/TI/RR/*N* = 5 ms/15°/300 ms/950 ms or 750 ms/33. The reference T1 was 280 ms. White Gaussian noise that represented a SNR of 10 and 15 was added to each simulated LGE signal. Here SNR is defined as the blood signal (blood T1 is 280 ms) divided by the standard deviation of the Gaussian noise. Two RR values, 750 ms and 950 ms, were used in the simulations to study the performance of the two methods under different heart rates. The standard deviation of the T1 estimates were calculated over the 10,000 simulations to study the precision of the method.

All phantom, animal and human studies were performed on a 1.5 T Siemens scanner (Siemens Healthcare, Erlangen, Germany).

### Phantoms

T1-refBlochi was validated in phantoms (Gd-doped water) consisting of 6 T1 s from 200 to 560 ms, which spanned the relevant range for post-contrast T1 values. The method was compared to a gold standard T1-mapping (spin echo inversion recovery, SE IR) method in a uniform head coil, with 6 TIs, and a TR = 3000 ms. The LGE protocol used for T1-refBlochi was TR/*α*/TI/RR/*N* = 3.8 ms/15°/300 ms/800 ms/37, 1 RR between inversions. The protocol was modified to explore sensitivity of T1-refBlochi to the protocol. Modifications in flip angle (changed to 10°), RR (changed from 900 to 700 ms), N (changed to 51), TR (changed to 5.4 ms) and underlying SNR (reduced by a factor of 4) were studied. The reference T1 for refBlochi was the shortest (194 ms).

### In vivo comparison of T1-refBLochi with multi-TI T1 mapping

We compared T1-refBlochi with a 3D multi-TI T1 mapping method, which is based on multiple LGE imaging, each with a different TI. The 3D multi-TI T1 mapping also provides an estimate of the combined proton density and T2* weighting, which was used to investigate its heterogeneity in vivo and its impact on T1-refBlochi accuracy. Four LGE measurements (with the same parameters as for refBlochi) were used in the multi-TI method, with three of them having different TIs (100,200 and 300 ms), and the last measurement having inversion disabled to mimic an infinite TI [11, 52]. For the three LGEs with different TI values, the steady-state longitudinal signal can be accurately modeled by Eq. 3. For the volume with no inversion pulse, however, the model is slightly different due to the absence of inversion pulse:

10 $$ {M}_{zss}\left({T}_1,{M}_0, RR,\alpha, TR,N\right)={M}_0\left(\frac{1-{pe}^{-\frac{RR}{T_1}}}{1-{\left(\mathit{\cos}\left(\alpha \right)\right)}^N{e}^{-\frac{RR}{T_1}}}\right) $$

A separate fitting of Eqs. 3 and 5 is needed for the inversion and non-inversion data, resulting in the following optimization problem:

11 $$ \mathit{\arg}\ \underset{T_1,{M}_0}{\mathit{\min}}\sum_{i=1}^3{\left({S}_i-{M}_0\left(\frac{1-2{e}^{-\frac{T{I}_i}{T_1}}+{pe}^{-\frac{RR}{T_1}}}{1+{\left(\mathit{\cos}\left(\alpha \right)\right)}^N{e}^{-\frac{RR}{T_1}}}\right)\right)}^2+{\left({S}_0-{M}_0\left(\frac{1-{pe}^{-\frac{RR}{T_1}}}{1-{\left(\mathit{\cos}\left(\alpha \right)\right)}^N{e}^{-\frac{RR}{T_1}}}\right)\right)}^2 $$

where *S*_1 − 3_ are the signals corresponding to the three TIs and *S*_0_ is the signal for the non-inversion LGE, respectively. The *M*_0_ and T1 maps were then generated voxel-by-voxel, using the Levenberg-Marquardt algorithm, implemented with MATLAB (MathWorks, MA, USA) Curve Fitting toolbox.

### Animal studies

Animal studies were performed in accordance with our Institutional Animal Care and Use Committee. All animals received humane care in compliance with the “Guide for the Care and Use of Laboratory Animals” published by the National Institutes of Health, the Animal Welfare Act, and Animal Welfare Regulations. Eight Yorkshire pigs were studied, including 3 control animals, and 5 animals imaged one to two weeks after infarction (average weight 36 ± 9 kg, average heart rate of 94 ± 16 bpm), with infarctions performed using a surgical left circumflex coronary artery occlusion or a 90 min balloon occlusion of the left anterior descending coronary artery.

Prior to CMR, animals were anesthetized, and ventilated. T1-mapping data was acquired starting from 20 to 30 min post-injection of 0.2 mmol/kg Gadobutrol (Bayer Healthcare). Three-dimensional LGE with 4 TIs (no inversion, 100,200, and 300 ms) were sequentially acquired, targeting both the LV and LA. 3D LGE used a fat-saturated, navigator-gated high resolution 3D inversion recovery spoiled gradient echo sequence, with ECG-triggering. Mid-diastole was chosen based on the RR interval. Spatial resolution was 1.2 × 1.2 × 3 mm^3^ before zero-filling, with FOV of 300 mm. TR/TE/*α*/*N* = 3.9 ms/1.9 ms/15°/37. Bandwidth was 490 Hz/pixel. The number of phase-encodings and slice-encodings was typically 256 and 18, respectively. No acceleration was used. One or 2 RR intervals between inversion pulses were used; two RR intervals were chosen for studies if the heart-rate was very high. The average interval between inversion pulses was 1010 ± 270 ms. Cartesian k-space sampling was used with centric reordering in the phase-encoding direction and sequential ordering in the slice-encoding direction. Navigator-gating used a ± 3 mm acceptance window, with no tracking. Data was acquired over an average of 14 ± 5 min (for four TI values). The data was processed with the multi-TI method to generate T1 maps and maps representing combined coil-sensitivity, T2* and proton density. These combined maps of coil-sensitivity, T2* and proton density, were analyzed to evaluate the variation of M_0_. Spatial registration between the four LGE images was not performed, but the quality of the generated T1 maps was visually good.

T1-refBlochi was performed with the LGE dataset acquired at TI = 300 ms. Blood was chosen for the single-point calibration and its T1 from the multi-TI method was used as the reference T1. The T1 maps generated from T1-refBlochi was compared to those from the multi-TI method, using visual inspection, and direct subtraction. For blinded comparison, ROIs were placed in blood, normal myocardium, and scar, on T1 maps from the multi-TI method, and automatically copied to the corresponding T1-refBlochi T1 map. The mean and standard deviation of T1 s were measured in each ROI, and compared.

### Patient studies

Five patients (3 males, age 53 ± 8) were imaged with 3D LGE covering the whole heart, about 20 min post 0.2 mmol/kg injection of gadolinium contrast agent, and a 2D 4-chamber ShMOLLI was acquired 5 ± 2 min later, on a Siemens 1.5 T scanner (Siemens Aera, Erlangen, Germany), using our clinical protocol: 360 mm × 270 mm FOV, 1.4 × 2.8 × 6 mm^3^, with 92 phase-encodings, and TR/TE/ *α* =2.6 ms/1.3 ms/35°. 3D LGE scan parameters were: FOV 380 mm × 498 mm, spatial resolution 1.5 × 1.5 × 3.6 mm^3^ before zero-filling, TR/TE/ *α* =3.9 ms/1.7 ms/15°, *N* = 27–53, RR = 904 ± 57 ms, TI = 287 ± 12 ms, and receiver bandwidth 360 Hz/pixel. The number of phase-encodings and slice-encodings was typically 336 and 44, respectively. Parallel imaging was used with GRAPPA factor of 2. The 3D k-space was sampled with centric reordering in the phase-encoding direction and sequential ordering in the slice-encoding direction. Navigator-gating was used with a ± 3 mm acceptance window, placed after the acquisition window in the mid-diastole. The 3D LGE image was transformed into a T1 map using refBlochi, and compared to ShMOLLI. Blood with T1 provided by ShMOLLI was used as the reference for refBlochi. The patient study was approved by our institutions IRB and all patients provided informed written consent. The patients were imaged for assessments of atrial fibrillation (*n* = 4) and hypertrophic cardiomyopathy (*n* = 1). ROIs were placed in the blood and myocardium, with mean T1 values compared between the two methods.

Using the left atrial post-contrast T1 maps in the patient, the extracellular volume fraction was approximated (ECVa) as:

12 $$ ECVa=\left(\left(\frac{1}{T{1}_m}\right)-\left(\frac{1}{T{1}_{m0}}\right)\right)\bullet \left(\frac{\left(1- HCT\right)}{\frac{1}{T{1}_b}-\frac{1}{T{1}_{b0}}}\right) $$

With an assumed T1_m0_ = 1150 ms, and a T1_b0_ = 1500 ms, and HCT = 0.45. T1_b0_ and HCT can be measured in each patient, but left atrial T1_m0_ is highly challenging to measure, due to e.g. registration error, additional scan time, and blood-myocardial partial voluming. Therefore, an approximation of ECV with presumed native T1, defined in Eq. 12, was used, and the sensitivity of ECV to native myocardial T1 was explored to estimate the approximation error. A plot of ECV for three post contrast blood T1 s and a post-contrast myocardial T1 of 300 ms shows the weak dependence on T1_m0_ (Additional file 1). A highly realistic scenario in which the native T1 in regions of edema are elevated by 100 ms [53, 54], from 1150 to 1250, results in an absolute deviation of ECV by +0.02 (+2%), when ECV is measured with T1_b_ = 350 ms, for any myocardial post-contrast T1. This absolute increase in ECV by 2% cannot be measured if T1_m0_ is assumed constant at 1150 ms. Therefore, ECVa is a reasonable and practical approximation of true 3D ECV.

## Results

### Simulations

**Fig. 2:**
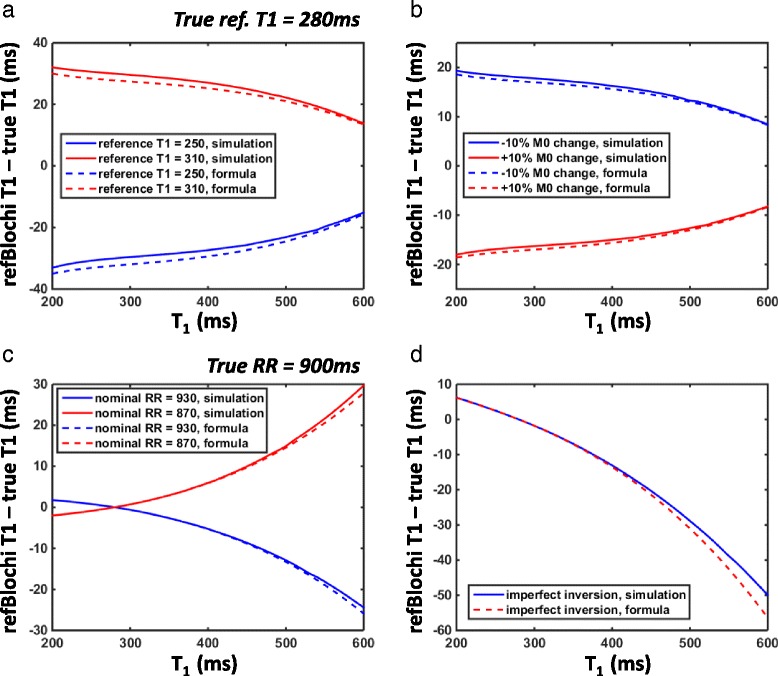
T1 estimate bias caused by four major sources of error: biased reference T1 estimate (**a**), proton density and T2* spatial variation (**b**), biased estimate of effective RR duration (**c**), and imperfect inversion (**d**). The solid lines represent the bias simulated from generating LGE signal and estimating T1 using T1-refBlochi. The dash lines represent the bias calculated by the analytical approximation formula

Figure 2 shows the T1-refBlochi bias caused by the four major sources of error. The error calculated based on the mathematical formulae (dash lines) excellently matched the simulated error (solid lines) in all cases, demonstrating the accuracy of the formulae. Error in the M_0_ estimate (Category 1) caused by either an inaccurate T1 reference or M_0_ heterogeneity, results in a bias that decreases with increasing T1 (Fig. 2a & b). For biased reference T1 estimate (±30 ms, Fig. 2a), the error for all T1 s longer than the reference T1 is less than the error in the reference T1. Therefore, choosing a short reference T1 reduces the bias present in the large T1 species. Fig. 2b shows the bias caused by a ± 10% change of M_0_ relative to the reference M_0_. The bias is within 20 ms, showing that T1-refBlochi is very robust against a mild spatial variation of M_0_. Error in the LGE signal modeling (Category 2) causes bias which increases with longer T1 s (Fig. 2c & d). Fig. 2c shows the bias of T1 estimate with an incorrect nominal RR (true RR ± 30 ms). The resultant bias is within 30 ms. Fig. 2d shows the bias of T1 estimate under worst-case imperfect inversion (inversion factor = 0.92). The bias is within 50 ms.

### Precision of T1-refBlochi

**Fig. 3:**
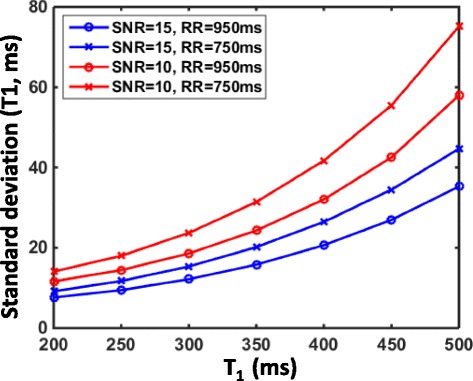
The standard deviation of the T1 estimates for T1 ranging from 200 ms to 500 ms for two different RR durations (750 ms and 950 ms) and two different blood SNRs (15 and 10). Precision is lower for longer T1 s and lower SNR

The result of the Monte-Carlo simulations is shown in Fig. 3. The standard deviation of the T1 estimates increases as T1 increases, consistent with the theoretical analysis. The largest standard deviation at T1 = 500 ms was 35 ms and 45 ms at SNR = 15 and 58 ms and 76 ms at SNR = 10 for RR of 950 ms and 750 ms, respectively. A longer RR is favorable but the difference due to variation of RR is rather small.

### Phantom validation

**Fig. 4:**
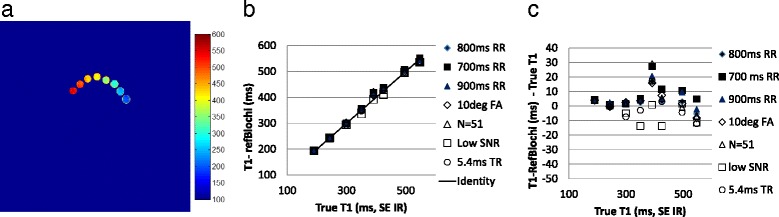
The refBlochi T1 map of the phantom (**a**) and the comparison between refBlochi T1 and the true T1 values shows excellent agreement (R^2^ = 0.99) (**b**,**c**). Seven scan protocols were tested, and T1 s ranged from 200 to 560 ms. The basic scan protocol used TR/*α*/N/RR/TI of 3.8 ms/15°/37/800 ms/300 ms. Then N, TR, SNR, RR interval and *α* were varied. Measurement of bias and standard deviation for refBlochi demonstrated good accuracy (−3 ± 9 ms) (**c**)

In phantoms, T1–refBlochi showed excellent agreement with the gold standard (SE IR) over multiple protocols (Fig. 4). Over all protocols and T1 ranges, the mean bias and standard deviation was -3 ms ± 9 ms and the correlation coefficient between T1-refBlochi and SE IR was R^2^ = 0.99. For the multi-TI comparison method, the mean bias and standard deviation was -8 ms ± 13 ms, and R^2^ = 0.99, compared to SE IR.

### Animal studies

#### Coil-sensitivity detrending

**Fig. 5:**
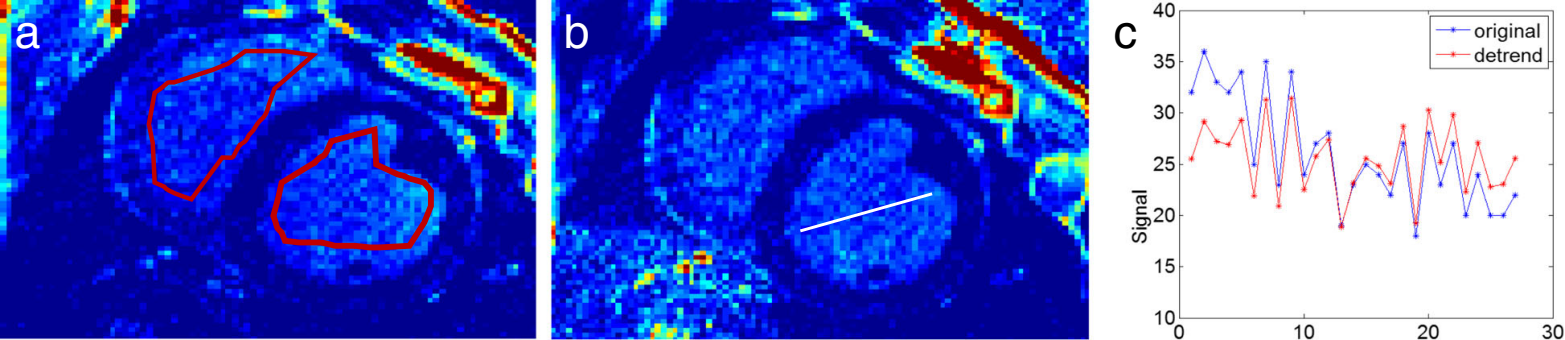
A representative slice before (**a**) and after (**b**) the 3D detrending algorithm. The *red* contours in (**a**) represent the non-contiguous ROIs used in the algorithm. **c** shows the effect of detrending along a reference line (*white line*) shown in (**b**)

Figure 5 shows the performance of the 3D detrending to remove coil-sensitivity effects for T1-refBlochi. Fig. 5a shows an LGE image before detrending, with non-contiguous ROIs drawn in each slice, outlining areas of the blood pool. The blood pool signal is expected to be isointense, except for coil sensitivity. These ROIs are used to detrend the image. Fig. 5b shows the slice after detrending. The removal of coil-sensitivity is evident in the line profiles across the blood pool (Fig. 5c), which demonstrate a decreased global slope after detrending.

#### T1-mapping

**Fig. 6:**
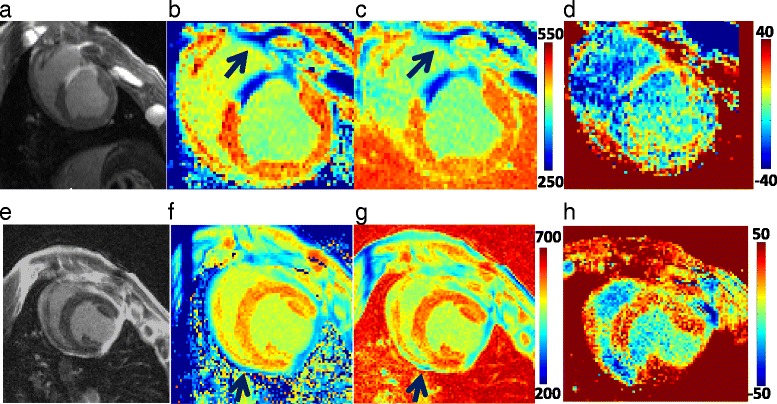
Representative T1 maps from 2 swine with myocardial infarction, with true spatial resolution of 1.2 × 1.2 × 3 mm^3^. **a**, **e** LGE images. T1maps using (**b**, **f**) MultiTI, and (**c**, **g**) refBlochi. **d**, **h**) The T1 difference images. In (**a**-**d**), note the RV scar (*arrows*) and the visible RV trabeculation. In (**e**-**g**), note the pericardial enhancement (*arrow*) which might not be visible with 2D methods

**Fig. 7:**
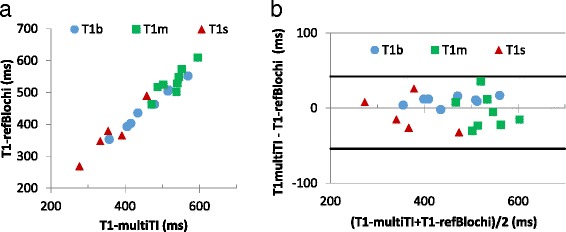
The T1 s of LV myocardium (T1 m), blood (T1b) and LV scar were compared (T1 s) on the multi-TI and T1-refBlochi maps in each swine. There was an excellent correlation (R^2^ = 0.96, slope = 0.98 between these T1 s (**a**), with Bland-Altman limits of agreement given as bias ±2SD of −6 ± 44 ms (**b**), excluding the blood pool T1 s which acted as the “reference” for refBlochi

Figure 6 shows data from two swine with myocardial infarction. A selected slice from the LGE volume shows the scar (A,E). The multi-TI method (B,F) and T1-refBlochi (C,G) maps are compared with identical scales. The difference images of the T1 maps for the two methods (D,H) show good agreement in the T1 measurement. Fig. 7 compares measured T1 s in ROIs placed identically in the left ventricle on the multi-TI method and T1-refBlochi maps, comparing T1 s in myocardial, blood and scar ROIs. There was an excellent correlation (R^2^ = 0.96, slope = 0.98) between these T1 s, with Bland-Altman limits of agreement given as bias ±2SD of −6 ± 44 ms, excluding the blood pool T1 s which acted as the “reference” for refBlochi (and were highly similar). Using large blood pool ROIs and a septal myocardial wall ROI, the average standard deviation of T1 was measured as 19 ± 9 ms and 35 ± 27 ms for blood T1 s and myocardial T1 s respectively. This accords well with the simulations of Fig. 3, showing that the precision of T1 measurement is reduced at longer T1 s but is overall adequate.

#### Proton density images, including residual coil-sensitivity and T2*-weighting (M_0_)

**Fig. 8:**
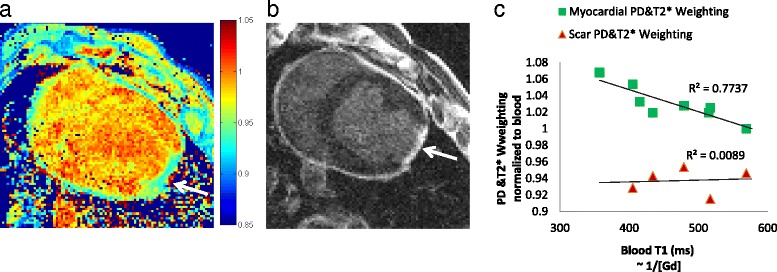
**a** A representative image of the proton density and T2* weightings in one pig, shown with the matching slice from the LGE volume (**b**). The map in (**a**) was normalized to the average weighting for blood. Note that the region with scar demonstrates a slightly lower weighting (~0.93). **c** A plot of the PD and T2* weighting ratios for each subject for myocardium to blood and scar to blood ratios, plotted vs. blood T1. The PD and T2* weighting had a mean scar/blood ratio of 0.94 ± 0.01 and a mean myocardium/blood ratio of1.03 ± 0.02

Figure 8a shows an example of the remaining weightings in the image, representing any coil-sensitivity, T2* and proton density but no T1-weighting. The combined weighting maps were obtained from the multi-TI method. The weighting is relatively homogeneous. Over all subjects, the average ratio of signal for scar/blood was 0.94 ± 0.01 (*p* = 0.10) and for myocardium/blood was1.03 ± 0.02 (*p* = 0.002) in the map.

#### Patient study

**Fig. 9:**
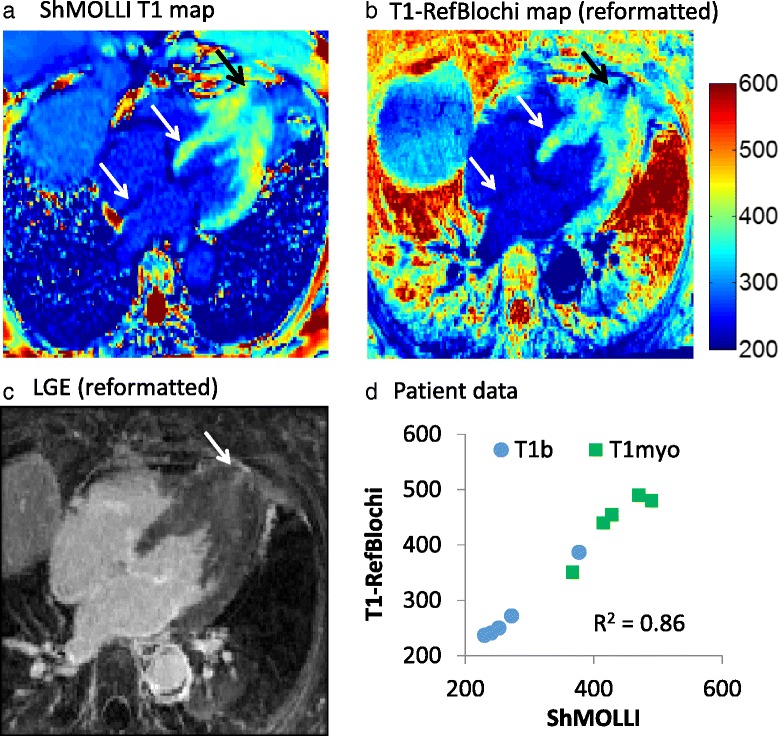
Post-contrast T1-maps with ShMOLLI, and refBlochi, acquired consecutively, in a hypertrophic cardiomyopathy patient. **a** 4-Chamber ShMOLLI. **b** Reformatted 3D refBlochi. Colormap is identical. The blood T1 (240 ms) in ShMOLLI was the reference T1. Note the similar T1 s of the myocardium, valves, and scar (*arrows*). The reformatted refBlochi T1 map provides more detailed view of the apical infarct. **c** The reference 3D LGE reformatted in 4 chamber view shows the scar (*white arrow*). **d** Measured myocardial and blood T1 from all five patients, with Bland-Altman limits of agreement given as bias ±2SD of −9 ± 42 ms

**Fig. 10:**
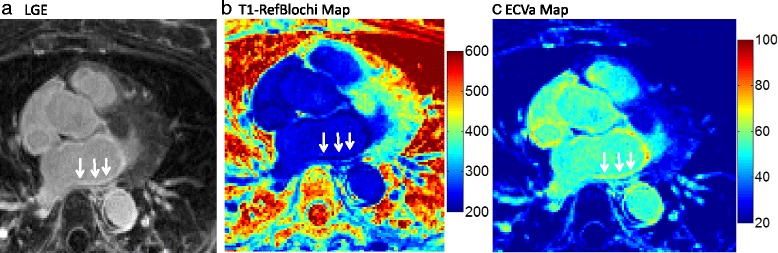
**a** 3D LGE showing a slice of the atrium, showing ubiquitous atrial fibrosis. Arrows point to fibrosis of the posterior wall. **b** The refBLochi T1 map. **c** An ECV estimate, obtained by assuming a standard native T1 (ECVa). The ECVa of the evident fibrosis was measured to be ~62% (i.e. diffuse fibrosis)

Figure 9 (a-c) compares the 3D reformatted T1-refBlochi map with the ShMOLLI T1 map, obtained in a 4-chamber view, in one patient. The reformatted LGE is shown for reference. In the five patients studied, the mean T1 bias ±1SD in myocardium and blood was -9 ms ± 21 ms, with a correlation of R^2^ = 0.86 (Fig. 9d). The similarities in the T1 maps are evident, showing the feasibility of this method in vivo. In the hypertrophic cardiomyopathy patient, left atrial fibrosis is evident on a 3D LGE slice (Fig. 10a). The T1 map shows a shortened T1 of the left atrial wall (Fig. 10b), and the ECVa is elevated (Fig. 10c, ~62%).

## Discussions

In this work, we demonstrate a novel method for 3D high resolution T1-mapping. T1-refBlochi uses only a single TI, and transforms a 3D high resolution LGE image to a T1 map of equal resolution directly. This technique is easy to apply clinically, since the clinician only needs to prescribe a 2D T1 mapping sequence immediately before or after the 3D LGE. The method assumes that proton density and T2* effects are small, and must remove the coil-sensitivity effects. A single-point calibration is then performed, based on the 2D T1 mapping, to estimate the combined weighting of proton density and T2*. T1 is then estimated voxel-by-voxel based on the one-to-one relationship between the signal and T1 for a specific LGE protocol.

Several limiting factors of the technique were analyzed, including biased estimation of the reference T1, residual effect from proton density, T2*, and coil sensitivity, effective RR deviation, and imperfect inversion. A small bias can arise in estimates of the reference T1, e.g., due to limitations of the 2D T1 mapping method or T1-drift between the application of 2D T1 mapping and LGE. The resultant error is no greater than the error in the reference T1. There is heterogeneity of M_0_ (proton density, T2*, and coil sensitivity) but it is small (<10%) based on previous literature and our in vivo study. Therefore, the bias of refBlochi due to heterogeneity of M_0_ is also small, e.g. about 20 ms for 10% error in M_0_. Other effects, such as the deviation of the effective RR and imperfect inversion, can be largely improved by a better design of the LGE sequence, e.g., recording RR duration of the k-space center readout for a more accurate RR estimate, using an inversion RF pulse with a higher inversion factor [51]. Overall, our data shows that the accuracy of T1-refBlochi should be comparable to other 2D cardiac T1 mapping methods, such as MOLLI. The precision of T1-refBlochi is good for shorter T1 s, i.e. scar and blood, but somewhat worse for longer T1 s and when blood SNR is lower. Therefore, methods which increase or maintain SNR are important. Other potential limiting factors include blood flow and magnetization transfer [14]. Blood flow is a problem for nearly all T1 mapping methods. Magnetization transfer causes incomplete inversion and over-saturation after a bSSFP or GRE echo train [14, 55]. While the incomplete inversion is common to all inversion-recovery based T1 mapping methods, the GRE readout used by T1-refBlochi is expected to be less sensitive to magnetization transfer due to use of smaller flip angles.

T1-refBlochi has several advantages over many 2D cardiac T1 mapping methods. First, many 2D T1 mapping techniques have a sensitivity to T2 and off-resonance due to usage of a bSSFP readout. T1-refBlochi uses GRE readout. This is important for the left atrium, where strong off-resonance is known to exist due to the pulmonary veins [56, 57]. Moreover, T1-refBlochi is not sensitive to heart rate, because of the usage of the Bloch equations, and based on our simulations (not shown), refBlochi is not affected greatly by errors in GRE flip angles, with less sensitivity than MOLLI. T1-refBlochi does not require de-rectification of the signal, and its precision is preserved because it is a single parameter fit. Most importantly, due to the high resolution inherent to 3D LGE, partial-voluming is largely reduced, making T1 mapping of RV and atrium possible.

To our knowledge, single-TI T1 mapping has not been explored before. In a general setting, this is not possible since the combined effect of proton density, T2*, and coil sensitivity is difficult to model. For cardiac applications, we found that this technique is feasible. First, the variation of proton density and T2* are small between scar, blood, diffuse fibrosis, and myocardium. Second, coil sensitivity can be removed by either coil sensitivity measurement or post-processing. Lastly, the T1 estimate is relatively insensitive to heterogeneity of M0. Considering the paramount importance of efficiency in 3D T1 mapping, a small tradeoff of the accuracy with a drastic improvement of efficiency is worthwhile.

The T1-refBlochi method has tremendous potential to improve interpretation of atrial LGE. For patients who have atrial fibrosis, these T1 maps will reflect the diffuse fibrosis that is enhanced in LGE. From these maps, an estimate of ECV (ECVa) can also be obtained, assuming standard pre-contrast T1 s. Approximate ECV maps are feasible because the “error in ΔR1 used for ECV calculation is less affected by errors in the pre-contrast T1” [14] (Additional file 1). ECV mapping is a more quantitative method for characterizing the burden of atrial fibrosis, compared with volumetric measurements of enhanced tissue, based on a signal threshold. For atrial LGE studies in particular, the critical T1 values (i.e. for scar) are those <T1 of blood, so refBlochi is highly suitable. Current quantification of atrial fibrosis requires a threshold choice for fibrosis segmentation [58]. However, this results in segmentation which depends on TI choice—itself affected by factors such as the presence of ventricular diffuse fibrosis—and by timing post-injection, and by the overall SNR of the study. T1/ECV mapping could provide more robust quantification.

In prior studies, 3D myocardial T1 mapping methods were validated using phantoms or common 2D techniques, such as MOLLI or SASHA. These 2D comparison methods are not appropriate for this study, mainly because they cannot provide the information regarding the spatial uniformity of combined proton density and T2* weighting in the heart region, which is a premise of T1-refBlochi. Therefore, we chose to use a 3D multi-TI LGE method, which we first validated using phantoms, at multiple heart-rates and scan parameters. The 3D multi-TI method is sufficiently straightforward in its framework, that is should perform similarly to any other multi-TI method [26, 32, 59], and not differ in performance too much when used in phantoms or in vivo. The 3D multi-TI method provides a direct quantification of the combined proton density and T2* weighting using curve fitting. The comparison between multi-TI and single-TI refBlochi testifies the accuracy and precision of the latter method.

There are several limitations associated with T1-refBlochi and this study. Firstly, it’s challenging to use T1-refBlochi for pre-contrast T1 mapping, thus only an approximate ECV can be calculated by using the method. A validation of the approximate ECV using histology is lacking. Secondly, in the animal study, the multi-TI LGE method is influenced by the T1-drift over time, roughly 3 ms per minute for blood at 10–20 min post injection of 0.2 mmol/kg of gadolinium contrast agent [60, 61]. Moreover, direct comparison of T1-refBlochi to MOLLI in swine was lacking. Thirdly, the technique was only validated in a small number of patients. Although this method has important applications in the RV and the atrium, our in vivo validation was limited to the LV. A larger patient study is highly warranted to study the value of the technique to quantify left atrial remodeling. Finally, the voxel size is limited, so that the LGE, and therefore the T1map is influenced by partial-voluming of blood and atrial wall. Black blood LGE methods can be used to mitigate the impact of blood [62, 63].

## Conclusions

In conclusion, we present a novel 3D high resolution cardiac T1 mapping method that requires only a single 3D LGE measurement and a reference T1. The method provides excellent efficiency, good accuracy, and adequate precision. The high resolution of the technique enables T1 mapping of atrial and RV wall, which is generally not possible with most 2D T1 mapping methods.

## Appendix A

After the *α* pulse train is applied, the longitudinal magnetization first regrows, and then is inverted after the next ECG trigger, followed by another regrowth period during the TI. The magnetization *M*_*zss*_ immediately before the *α* pulse train, is therefore given by

A1 $$ {M}_{zss}=-\left({M}_z^{+}{e}^{-\frac{RR- TR\cdotp N- TI}{T_1}}+{M}_0\left(1-{e}^{-\frac{RR- TR\cdotp N- TI}{T_1}}\right)\right){e}^{-\frac{TI}{T_1}}+{M}_0\left(1-{e}^{-\frac{TI}{T_1}}\right)=-{M}_z^{+}{e}^{-\frac{RR- TR\cdotp N}{T_1}}+{M}_0\left(1-2{e}^{-\frac{TI}{T_1}}+{e}^{-\frac{RR- TR\cdotp N}{T_1}}\right) $$

where N is views per segment, *M*_0_ is the equilibrium magnetization, RR is the R-R interval duration, TR is the spacing between two *α* pulses, RR-TR∙N-TI is time between the end of the *α* pulse train and the next inversion pulse. ${M}_z^{+}$ is the transient signal of FLASH after N *α* pulses, which is related to the magnetization just before the *α* pulse train (Mzss) as [35]:

A2 $$ {M}_z^{+}={M}_{zss}{\left(\cos \left(\alpha \right)\right)}^N{e}^{-\frac{TR\cdotp N}{T_1}}+\frac{1-{e}^{-\frac{TR}{T_1}}}{1-\cos \left(\alpha \right){e}^{-\frac{TR}{T_1}}}\left(1-{\left(\cos \left(\alpha \right)\right)}^N{e}^{-\frac{TR\cdotp N}{T_1}}\right){M}_0 $$

Substituting equation (A2) into (A1), we obtain the following equation for the steady-state *M*_*zss*_ in the late enhancement sequence.

A3 $$ {M}_{zss}=-{M}_{zss}{\left(\cos \left(\alpha \right)\right)}^N{e}^{-\frac{RR}{T_1}}+{M}_0\left(1-2{e}^{-\frac{TI}{T_1}}+{pe}^{-\frac{RR}{T_1}}\right) $$

where.

Rearranging the terms in equation (A3), we obtain an analytical expression for the steady-state *M*_*zss*_.

A5 $$ {M}_{zss}=\frac{M_0\left(1-2{e}^{-\frac{TI}{T_1}}+{pe}^{-\frac{RR}{T_1}}\right)}{1+{\left(\cos \left(\alpha \right)\right)}^N{e}^{-\frac{RR}{T_1}}} $$

## Appendix B

1. The following derivation is for Category 1, which includes all error sources that cause an inaccurate estimate of M_0_ (i.e. heterogeneity due to coils, proton density, and T2*). Let ${\widehat{M}}_0$ represent the estimate of *M*_0_ from the single-point calibration, and ${\widehat{T}}_1$ the estimate of *T*_1_ based on T1-refBlochi. Because the LGE signal is constant, any error in M_0_ is absorbed as an error in the estimate of T_1_:

B1 $$ {M}_0f\left({T}_1\right)={\widehat{M}}_0f\left({\widehat{T}}_1\right) $$

where the left side of the equation is the LGE signal acquired for each T1, and the right side is the model that contains the parameter ${\widehat{T}}_1$ that we need to estimate based on the equation. Let $\delta ={\widehat{M}}_0/{M}_0$ represent the relative amount of bias in M_0_ estimate and $\Delta {T}_1={\widehat{T}}_1-{T}_1$ the bias present in the T1 estimate. We then have

B2 $$ f\left({T}_1\right)=\delta f\left({T}_1+\Delta {T}_1\right)\approx \delta \left(f\left({T}_1\right)+{f}^{\prime}\left({T}_1\right)\Delta {T}_1\right) $$

based on the first-order Taylor expansion. Solving Equation (B2) with respect to Δ*T*_1_, we have

B3 $$ \Delta {T}_1\approx \frac{\left(1-\delta \right)f\left({T}_1\right)}{\delta {f}^{\prime}\left({T}_1\right)} $$

2. The following derivation is for category 2, which includes all error sources that cause an inaccurate modeling of the LGE signal. Let *f* ^true^(·) represent the true LGE modeling function, i.E., the true LGE signal is generated by *M*_0_ *f* ^true^(*T*_1_). T1-refBlochi, on the other hand, uses an approximated modeling function based on equation (5). The reconstruction solves the following equation:

B4 $$ {M}_0{f}^{\mathrm{True}}\left({T}_1\right)={\widehat{M}}_0f\left({\widehat{T}}_1\right)\approx \delta {M}_0\left(f\left({T}_1\right)+{f}^{\prime}\left({T}_1\right)\Delta {T}_1\right) $$

which leads to

B5 $$ \Delta {T}_1\approx \frac{f^{\mathrm{True}}\left({T}_1\right)-\delta f\left({T}_1\right)}{{\delta f}^{\prime}\left({T}_1\right)} $$

## Additional file

Additional file 1: ECV measured for 3 post-contrast blood T1 s, and a post-contrast myocardial T1 of 300 ms, as a function of native myocardial T1. ECV is relatively insensitive to variation of native myocardial T1 in all cases. (PDF 53 kb)

## Abbreviations

aECV: approximated ECV
bSSFP: balanced steady-state free precession
ECV: extracellular volume fraction
GRE: gradient echo
LA: left atrium
LGE: late gadolinium enhancement
LV: left ventricle/left ventricular
MOLLI: modified look-locker inversion recovery
RV: right ventricle/right ventricular
SASHA: saturation recovery single-shot acquisition
ShMOLLI: shortened modified look-locker inversion recovery
T1-refBlochi: the proposed 3D T1 mapping method that uses **ref**erence signal and **Bloch** equations
