# Supplementary figures and images for: T1-refBlochi: high resolution 3D post-contrast T1 myocardial mapping based on a single 3D late gadolinium enhancement volume, Bloch equations, and a reference T1
Source: J Cardiovasc Magn Reson. 2017 Aug 18;19:63. doi: 10.1186/s12968-017-0375-1 (PMC5563030; doi:10.1186/s12968-017-0375-1)

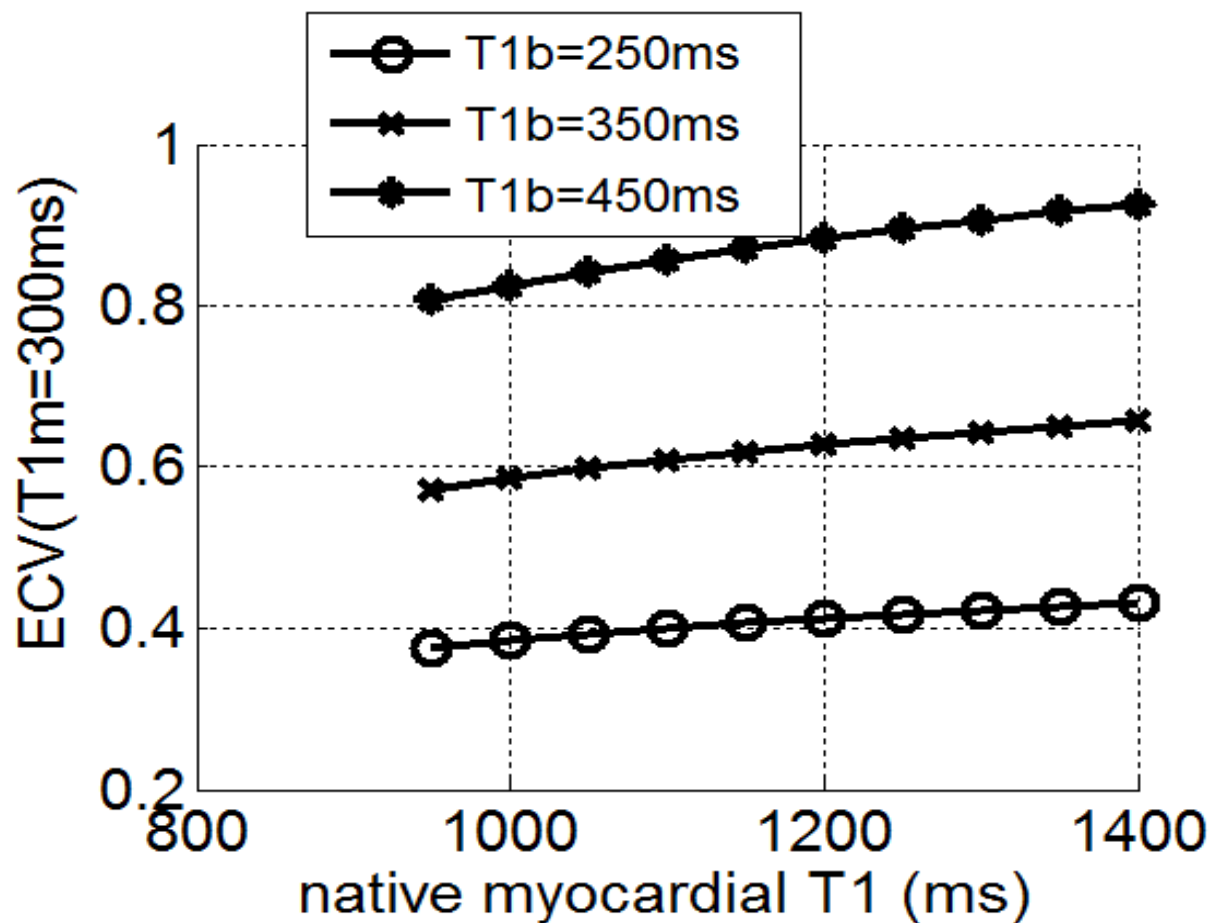

Supplement: Additional file 1: — ECV measured for 3 post-contrast blood T1 s, and a post-contrast myocardial T1 of 300 ms, as a function of native myocardial T1. ECV is relatively insensitive to variation of native myocardial T1 in all cases. (PDF 53 kb) [file 12968_2017_375_MOESM1_ESM.pdf]
